# Multifrequency dielectric mapping of fixed mice colon tissues in cell culture media *via* scanning electrochemical microscopy

**DOI:** 10.3389/fbioe.2023.1063063

**Published:** 2023-02-09

**Authors:** Varun Vyas, Niranjan G. Kotla, Yury Rochev, Anup Poudel, Manus Biggs

**Affiliations:** ^1^ CÚRAM, SFI Research Centre for Medical Devices, University of Galway, Galway, Ireland; ^2^ CNRS, LIEC, Université de Lorraine, Nancy, France

**Keywords:** AC-SECM, dielectric loss, tissue, multifrequency mapping, EIS, impedance, mice colon, tan (delta)

## Abstract

Alternating current scanning electrochemical microscopy (AC-SECM) is a powerful tool for characterizing the electrochemical reactivity of surfaces. Here, perturbation in the sample is induced by the alternating current and altered local potential is measured by the SECM probe. This technique has been used to investigate many exotic a range of biological interfaces including live cells and tissues, as well as the corrosive degradation of various metallic surfaces, etc. In principle, AC-SECM imaging is derived from electrochemical impedance spectroscopy (EIS) which has been used for a century to describe interfacial and diffusive behaviour of molecules in solution or on a surface. Increasingly bioimpedance centric medical devices have become an important tool to detect evolution of tissue biochemistry. Predictive implications of measuring electrochemical changes within a tissue is one of the core concepts in developing minimally invasive and smart medical devices. In this study, cross sections of mice colon tissue were used for AC-SECM imaging. A 10 micron sized platinum probe was used for two-dimensional (2D) tan δ mapping of histological sections at a frequency of 10 kHz, Thereafter, multifrequency scans were performed at 100 Hz, 10 kHz, 300 kHz, and 900 kHz. Loss tangent (tan δ) mapping of mice colon revealed microscale regions within a tissue possessing a discrete tan δ signature. This tan δ map may be an immediate measure of physiological conditions in biological tissues. Multifrequency scans highlight subtle changes in protein or lipid composition as a function of frequency which was recorded as loss tangent maps. Impedance profile at different frequencies could also be used to identify optimal contrast for imaging and extracting the electrochemical signature specific for a tissue and its electrolyte.

## Introduction

Scanning electrochemical microscopy (SECM) was first developed by Bard et al. (1989) and Engstrom and Pharr (1989). Almost 30 years later, it has proven to be a powerful tool for the detection of local variations in surface electrochemical properties. SECM is a scanning probe technique in which a small conductive probe is immersed in an electrolyte solution and is used for mapping a current response based on the surface topography and local electrochemical activity.

Various operational modes for mapping surface topography like constant height and constant distance, are described elsewhere (Bergner et al., 2013). Electrochemical mapping is typically carried out in DC amperometric mode, and redox species are added to the electrolyte, which produces current at the interface of a mobile micro-electrode. In close proximity to the surface of interest, fluctuation in the current provides valuable information regarding surface topography and its chemical composition (Lin et al., 2018; Li et al., 2022). Xia et al. (2019) used a similar technique where electrochemical noise from current or potential fluctuations can produce specific signatures related to the rupture of organic coatings. The Scanning Reference Electrode Technique (SRET) is another complementary technique to SECM that has also been used to study the corrosion resistance of alloys in the presence of specific organic molecules (Xia et al., 2015). Some groups have also developed soft micro-electrodes for identifying biomarkers in metastatic melanomas and injected nanomaterials in human tissue (Lin et al., 2016; Lin et al., 2017; Lin et al., 2018). SECM imaging can also be performed in AC mode. In alternating current scanning electrochemical microscopy (AC-SECM), changes in the impedance due to various faradaic processes provides a feedback signal similar to SECM imaging in DC mode. This high-resolution AC-SECM impedance mapping can be performed over a range of frequencies, and AC current flow during the measurement is induced by the superimposition of a sinusoidal waveform over a constant DC potential (Diakowski and Ding, 2007). An added advantage of this technique is that it does not require the addition of a redox mediator into the electrolyte solution (Li et al., 2022). The major advantage of this technique lies in the ability to identify microscopic domains with differential electrochemical activity which can be exploited to investigate biological surfaces (Polcari et al., 2016).

Though with SECM one can examine various types of surfaces, its use for dielectric loss tangent mapping of biological surfaces has not been reported in the literature. For the past three decades, Electrochemical impedance spectroscopy (EIS) has been used for measuring the electric and dielectric response of biological tissues over a wide range of frequencies and it is widely being explored as a non-invasive technique to augment medical device function (Kerner et al., 2002; Halter et al., 2007; Dean et al., 2008). The technique was first introduced in the late 19th century by Heaviside (1892). Since then various institutions have used this technique for monitoring fuel cells and batteries, for corrosion analysis and adsorption characteristics at an interface, and for the investigation of polymers, or coating over conductive surfaces (Ribeiro et al., 2015; Ribeiro and Abrantes, 2016; Leuaa et al., 2020; Wang et al., 2021). The dielectric properties of biological tissues were first fully described by Schwan et al. (1957). For analyzing properties of biological materials, dielectric properties are divided into three dispersions: *α*-Dispersions (10 Hz to kHz) that are associated with biological membranes, *β-*Dispersions (1 kHz to MHz) where encompassing the polarization of cellular membranes, proteins, and other molecules, and *γ-*Dispersions (≥10 GHz) where the polarization of water molecules can be observed. Within biological systems, a cellular membrane acts as a barrier to ion flow within the intra- and extracellular electrolytes. Such membranes are composed of lipids and proteins. Here, β dispersions can provide insight into bulk changes in tissue composition and physiological processes due to infection, injury, or a chronic disorder (Pethig, 1984). However, EIS is conventionally a macroscopic technique with a major disadvantage due to the large surface area of the electrode in contact with the tissue and the signal is an averaged output of the entire surface of the electrode that is exposed to the tissue. Most of the interesting processes occur at the cell membrane interface and interstitial spaces within a tissue. Hence, any minute variations in the impedance due to various molecular perturbations are not easily accessible from the overall impedance response of the tissue. This limitation of “global dielectric” *ex vivo* tissue analysis can be resolved by tissue impedance mapping at high spatial resolution using a microelectrode.

Dielectric mapping is mostly limited to Atomic Force Microscopy (AFM) where the image scan size is limited to approx. 10 microns. Recent investigations with Scanning Dielectric Microscopy (SDM) on synthetic cell membranes and purified proteins have relied on a modified Atomic Force Microscopy (AFM) approach where measurements were performed both in air and aqueous environment (Gramse et al., 2013; Lozano et al., 2018). This limits imaging capabilities when the sample of interest is a tissue section whose electrochemical profile needs to be mapped in an aqueous environment. Limited data is available on the description of large area dielectric loss tangent mapping of the tissue in cell culture media. SDM measurements are typically derived from Electrostatic Force Microscopy (EFM), where an oscillating probe measures surface potential and charge distribution. After extensive data processing, one can estimate dielectric constant (ε_r_) of the material of interest (Fumagalli et al., 2012). However, for most clinical applications dielectric loss tangent (tan δ ) is a more significant descriptor of tissue composition as opposed to the dielectric constant of the tissue (Fahmy et al., 2020; Manoufali et al., 2020). Tan δ is a direct measure of change or deviation from the standard biological state of the tissue. Popular electrochemical mapping methods provide us with the current and impedance profiles of the sample of interest. There is always some contribution from the surrounding media, which may not directly reflect protein denaturation and changes in the conformation of biomolecules that give defined structure to cells and tissue. Dielectric loss maps in the β dispersion range can give significant insight into biomolecular modifications like protein denaturation or cell death due to an underlying biological disorder.

This article introduces a complementary technique called Scanning Loss Tangent Mapping (SLTM), which is derived from AC-SECM-based impedance mapping. In this case, impedance-related information between a biological sample and a conductive probe in an ionic environment can be used to develop the tissue’s loss tangent (tan) profile. A non-oscillating probe at a fixed distance from the sample provides an impedance profile, and thereafter tan δ maps were developed at different frequencies. In this study, histological cross sections of the mouse colon were used as test biological material to develop a tan δ mapping approach as an indicator of dielectric absorption in complex tissues. Here, multifrequency impedance mapping (MIM) of the biological tissue was performed to segregate and identify various microclusters associated with dielectric loss signatures within a tissue cross-section. Signatures from various components within a tissue correspond to the regions with clearly defined boundaries when compared with the hematoxylin and eosin (H&E) stained section of the mice colon. Multifrequency scans revealed minute changes in the dielectric loss as a function of frequency. This gave a comprehensive overview of the dielectric loss within a tissue as compared to a standard EIS measurement where a probe needs to indent the tissue and a 2D loss tangent versus frequency profile of the tissue is generated. In the present work, we introduce three important aspects related to SLTM and MIM. First, tan δ mapping of the tissue under investigation. Second, multifrequency maps can be used to determine the optimal frequency for imaging in a highly ionic environment (i.e., cell culture media). And finally, multifrequency maps can help us understand evolution of tan δ signatures as a function of frequency.

## Experimental

### Mice colon cross section processing

Mice colon cross sectioned tissues were used for AC-SECM imaging. In brief, C57BL/6 strain mice colon tissues were fixed in 10% buffered formaldehyde, processed using an Excelsior AS Tissue Processor (Thermo Scientific), and embedded in paraffin. Paraffin embedded tissues were subsequently microtome sectioned (Thermo Scientific) at a thickness of 8 μm; two to three sections were placed on each slide, which were deparaffinized and hydrated using a standard lab protocol (xylene two washes-5 min each, ethanol 2 min each of 100%, 90%, and 70%). Sections were kept in 0.5% Periodic acid for 8 min in dark to oxidize glycogen to form aldehyde groups. Deparaffinized sections were subsequently stained with Haematoxylin for 5–10 min and washed with tap water for 2 min. Sections were further stained for 30 s with Eosin, and rinsed in running tap water, dehydrated in 100%, 70%, 50% ethanol 2 min each and cleared in xylene for 20 min and mounted with a cover slip, dried overnight, and imaged with light microscopy. The mice colon tissue sections used for the study were acquired from an approved study (committee id AE19125/P097) by the Animal Care Research Ethics Committee at the National University of Ireland, Galway, and the Health Product Regulatory Authority, Ireland.

### AC-SECM imaging

**FIGURE 1:**
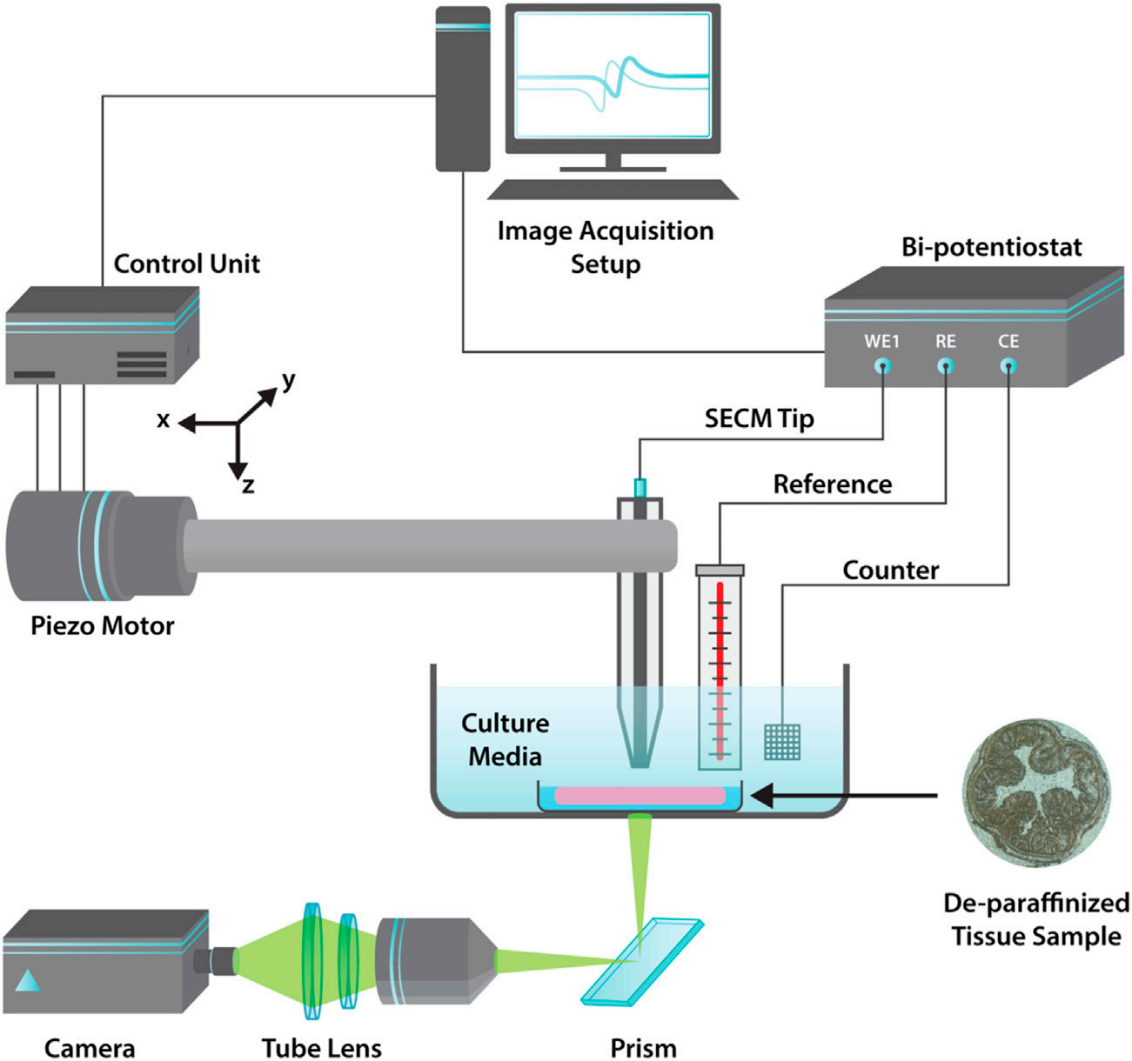
Graphical schematic illustration of the AC-SECM imaging apparatus with a three probe set-up used for dielectric mapping of colon tissues.

Constant height imaging was performed using a Biologic AC-SECM workstation (M-470) fitted with a SP-300 potentiostat. A 25 μm glass embedded Pt microelectrode with RG ratio of ∼10 was used as the scanning probe. A standard three electrode set-up, employing a working electrode (Pt microelectrode), reference electrode (AgCl) and counter electrode (Pt strip) was used for imaging (Figure 1). The spatial resolution was maintained at 30 micron with a DC bias of −0.25 V and AC bias of 2Vp-p. For dielectric mapping, whole colon cross section (CCS) were imaged at a frequency of 10 kHz and multifrequency imaging was performed at 100 Hz, 10 kHz, 300 kHz, 900 kHz. The scan rate for the imaging was kept at 150 μm/s . All imaging was performed in Dulbecco’s Modified Eagle Medium (DMEM) at room temperature. Image processing and data analysis were then performed using Matlab software.

### Theoretical background

In the field of dielectric physics, one can get valuable information about the conduction, polarization, and dielectric behaviour of any material. Permeability and permittivity can change with temperature, frequency, and the molecular structure of the material. The present study evaluates the dielectric behaviour of a highly conductive tissue sample in a cell culture media. Dissipation of electrical energy is measured as the dielectric loss tangent, which is also represented as tan δ . Dielectric loss is calculated from complex impedance data as a ratio of real (ReZ - $Z^{\prime }$ ) and imaginary (ImZ - $Z^{\prime \prime }$ ) impedance (Dean et al., 2008; Joshi et al., 2017) using the expression given below:

$$\varepsilon^{\prime }=\frac{t}{\omega A\varepsilon_{o}}.\frac{Z^{\prime \prime }}{Z^{\prime^{2}}+Z^{{\prime \prime }^{2}}},\varepsilon^{\prime \prime }=\frac{t}{\omega A\varepsilon_{o}}.\frac{Z^{\prime }}{Z^{\prime^{2}}+Z^{{\prime \prime }^{2}}},\tan\delta =\frac{\varepsilon^{\prime \prime }}{\varepsilon^{\prime }}=\frac{Z^{\prime }}{Z^{\prime \prime }}$$ (1)

where t , the distance between the electrodes; A , the cross-section area of the electrode, ω , the radial frequency; $\varepsilon_{o}$ , the permittivity of free space; $\varepsilon^{\prime }$ , the real permittivity, and $\varepsilon^{\prime \prime }$ , the imaginary permittivity.

For conductive biological samples in cell culture media, dielectric absorption is higher for the larger values of tan δ . At lower frequencies, electrical impedance in tissues is dominated by their resistive component, which increases their corresponding tan δ values (Schwan, 1992). Most of the hydrated proteins associated with tissues have large dipole moments arising from the conformation of the polypeptide backbone and ionizable acidic and basic side chains along their length (Pethig, 1984) and β dispersions between 10 kHz and 20 MHz are associated with a dielectric loss. Critically, tissue disease states can lead to denaturation of the protein modulating their dielectric loss properties through changes in associated water and ion mobility (Barringer and Bircan, 2006). Such effects can be observed over a range of frequencies using bulk EIS measurements or rendered into a dielectric loss map using high spatial resolution techniques such as SECM. Also, real and imaginary impedance data may not reflect the degree of dielectric loss between various biological components in tissue. With the novel approach of loss tangent mapping presented in this investigation, one can measure such minute fluctuations in β dispersions between cellular and extracellular elements in tissue sections.

## Results and discussion

### Dielectric mapping of colon cross sections

This investigation introduces the novel concept of tan δ profiling of C57BL/6 strain mice colon sections with a thickness of 8 μm by AC-SECM imaging. A standard approach curve method described previously was used to find the tissue surface (Eckhard and Schuhmann, 2008; Eckhard et al., 2009). The placement of the probe was confirmed using a custom built inverted optical setup, coupled to the M-470 workstation (Figure 1; Supplementary Figure S1). Imaging was performed over a large scan area of 4.41 mm^2^.

Before imaging, EIS frequency-sweep measurements were performed over sectioned colon tissues and adjacent amorphous SiO_2_ surface (glass slide). Here, the glass surface was uniformly coated with organic materials from the electrolyte (DMEM), therefore it gives a uniform signature around the tissue. In this article, the area surrounding the tissue will be referred to as the glass surface. Both impedance and tan δ measurements of these regions between 1 Hz and 1 MHz can be observed in Supplementary Figure S2. No significant difference in the impedance profiles of glass and the colon surfaces was observed at frequencies ranging from 1 Hz to 1 kHz. However, significant differences in tan δ measurements were observed as frequencies approached 10 kHz, as indicated in Supplementary Figure S2B. Subsequently, a frequency of 10 kHz was selected for dielectric mapping of the mice colon section. It is important to note that the frequency sweep data here was not representative of the whole tissue. During a surface frequency scan, the data collected as a function of frequency only provides information related to the region in the immediate proximity of the probe. In order to map changes in tissue impedance and related parameters as a function of frequency at the microscale, multifrequency scans across sectioned colon tissue were performed.

**FIGURE 2:**
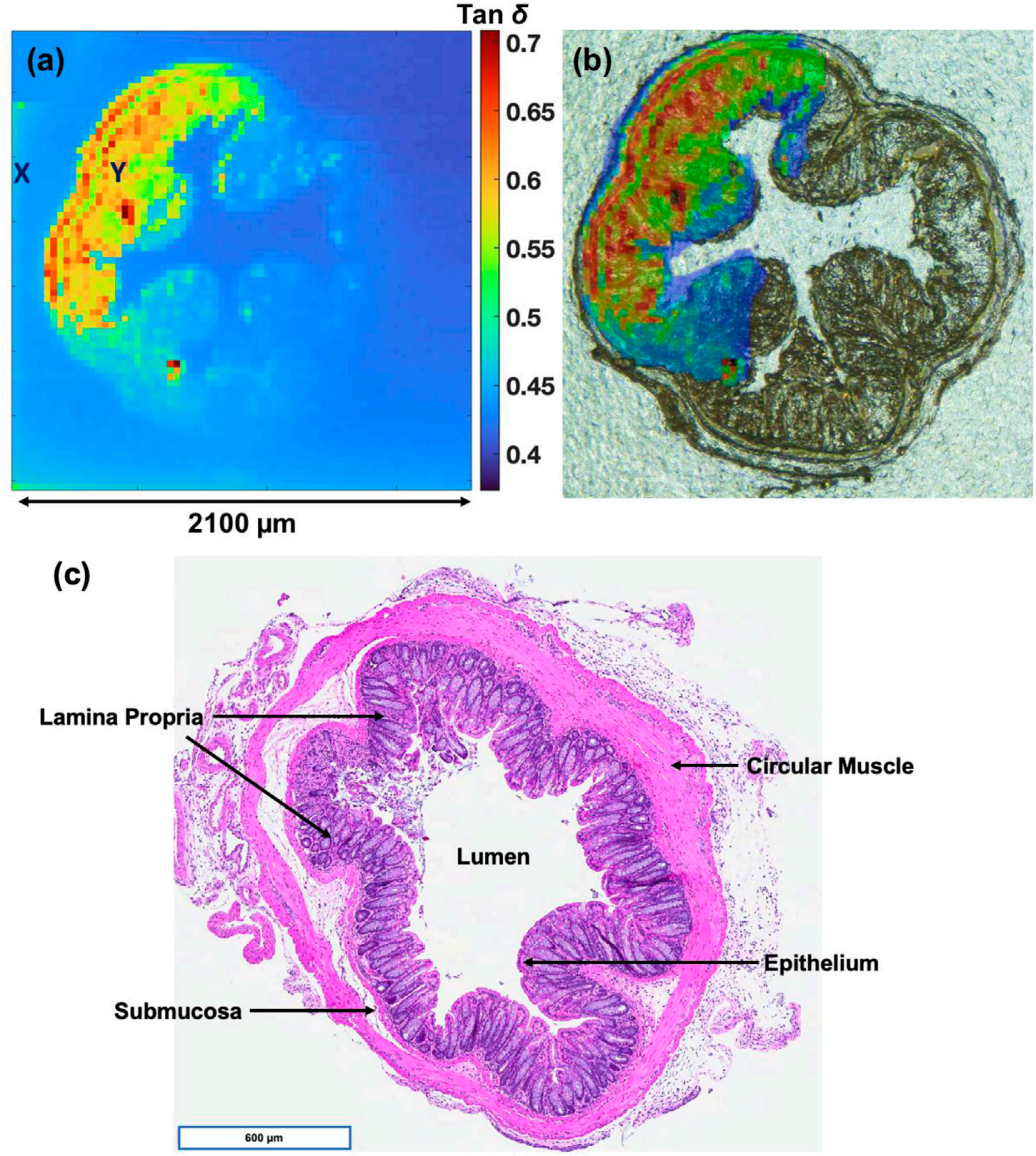
**(A)** Tan δ map of a deparaffinized mouse colon section. Frequency vs. impedance measurements were performed on the glass substrate (*X*) and colon section (*Y*). **(B)** An overlay of Tan δ and bright field image of the colon section. **(C)** H&E stained section derived from the same mice colon.

A representative dielectric loss map is shown in Figure 2A, and a corresponding bright field image of the same paraffinized section is given in Figure 2B. The tan δ map of the CCS was calculated as a ratio of real (ReZ) and imaginary (ImZ) maps. Tan δ was described earlier in Eq. 1. ReZ and ImZ surface scans of the colon are given in Supplementary Figures S3A, B), respectively: Here, SECM imaging was performed in constant height mode (Bergner et al., 2013). On closer inspection of Figure 2A, one can observe a non-uniform distribution of intensities from the top left corner of the image to the bottom right. During a large area scan in constant height mode, one may observe an intensity gradient that may arise due to a tilt in the substrate topography. During potentiometric measurements, as the separation distance between the probe and the surface increases, a suitable feedback signal required to measure the surface properties cannot be detected at a constant distance from the probe. Therefore, a small degree of tilt in the sample produces ambiguities in the measurement, especially over the region that is far from the surface. In Figure 2A, the distance between the probe and the CCS in the top left of the image was relatively less as compared to the bottom right side of the image. Hence, a loss of contrast can be observed in the bottom right of the image. When the scan size is greater than 1,000 μm any artefacts due to microscale tilt in the substrate cannot be corrected with the help of plane/tilt correction features available in various imaging software or by any mechanical means.

A large area scan of 2,100 µm^2^ was then overlaid with the bright field image of the mouse colon. This overlay revealed discrete differences in the dielectric properties of the colon section, Figure 2B. The degree of dielectric loss between the different regions in tissue reflects differences in local biochemistry and tissue density. The structural arrangement of different tissues within mice colon is marked and labeled in a corresponding H&E stained image derived from another section of the same mice colon, Figure 2C. Specifically, differences in the dielectric loss between the colon submucosa and circular muscle could be clearly observed. Here, the submucosa possessed a tan δ value of ∼0.55 and the circular muscle running along the outer periphery demonstrated a tan δ value of ∼0.65. The connective tissue of the submucosa consists of lymphatic and large blood vessels, as well as nerves that branch into the muscularis externa and the mucosal layer forming the colon lumen. Smooth muscle forms a circular muscle layer near the outer periphery of the colon (Boudry et al., 2004). It can be inferred that the dense layer of smooth muscle, which has a rich network of actin and myosin filaments produces a higher dielectric loss in the presence of an external electric field. Conversely, the connective tissue forming the submucosa possessed a less dense conformation that reduced the measured dielectric loss. The colon lamina propria (Figures 2A, C) demonstrated a relatively larger variation in dielectric loss (∼0.5–0.7). This tissue forms the least dense regions of the mucosa and contains predominantly collagen and elastin as well as non-connective tissue components, i.e., blood and lymphatic vessels (Boudry et al., 2004).

### Multifrequency mapping of colon sections

In this study, we performed a multifrequency analysis of the colon sections as a means to identify the optimal frequency for high contrast imaging. Furthermore, to facilitate biological imaging, cell culture media was used as the imaging electrolyte. Such media contain a high concentration of ions, amino acids, and other organic molecules. Critically, the majority of previous theoretical work and modelling of biological tissues has been focused on imaging under dilute ionic solutions, and AC-SECM surface scans are rarely conducted above 100 kHz, necessitating a preliminary study into the identification of the optimal frequency for cell-media based imaging.

Variations in electrochemical mapping can be induced by a number of factors, such as electrolyte/cell culture media, temperature fluctuation or impedance scanning frequency. Most commercial AC-SECMs can perform impedance mapping over a wide range of frequencies (1 Hz to 1 MHz). However, for most biological studies, SECM imaging is performed within the 100 kHz range, which ensures optimal tissue relative permittivity through ionic diffusion processes at the cell membrane, associated with low frequency α dispersions (Raicu and Feldman, 2015). The tissue sections used in this study had a thickness of 10 micron and were mounted onto an insulating glass (amorphous SiO_2_) surface. At a fixed temperature and frequency, the only factor that may have a significant influence is the composition of the electrolyte. The basic theory that explains ionic conduction in an electrolyte can be divided into three sets of problems. First, ultrafast solvation of ions, which affects the magnitude of dielectric friction. The second problem takes into account the concentration dependence of ionic conductance, and finally, the motion of ions in the presence of an oscillating electric field. The dynamics of ionic relaxation in an electrolyte are described elsewhere (Chandra et al., 1993; Chandra and Bagchi, 2000).

**FIGURE 3:**
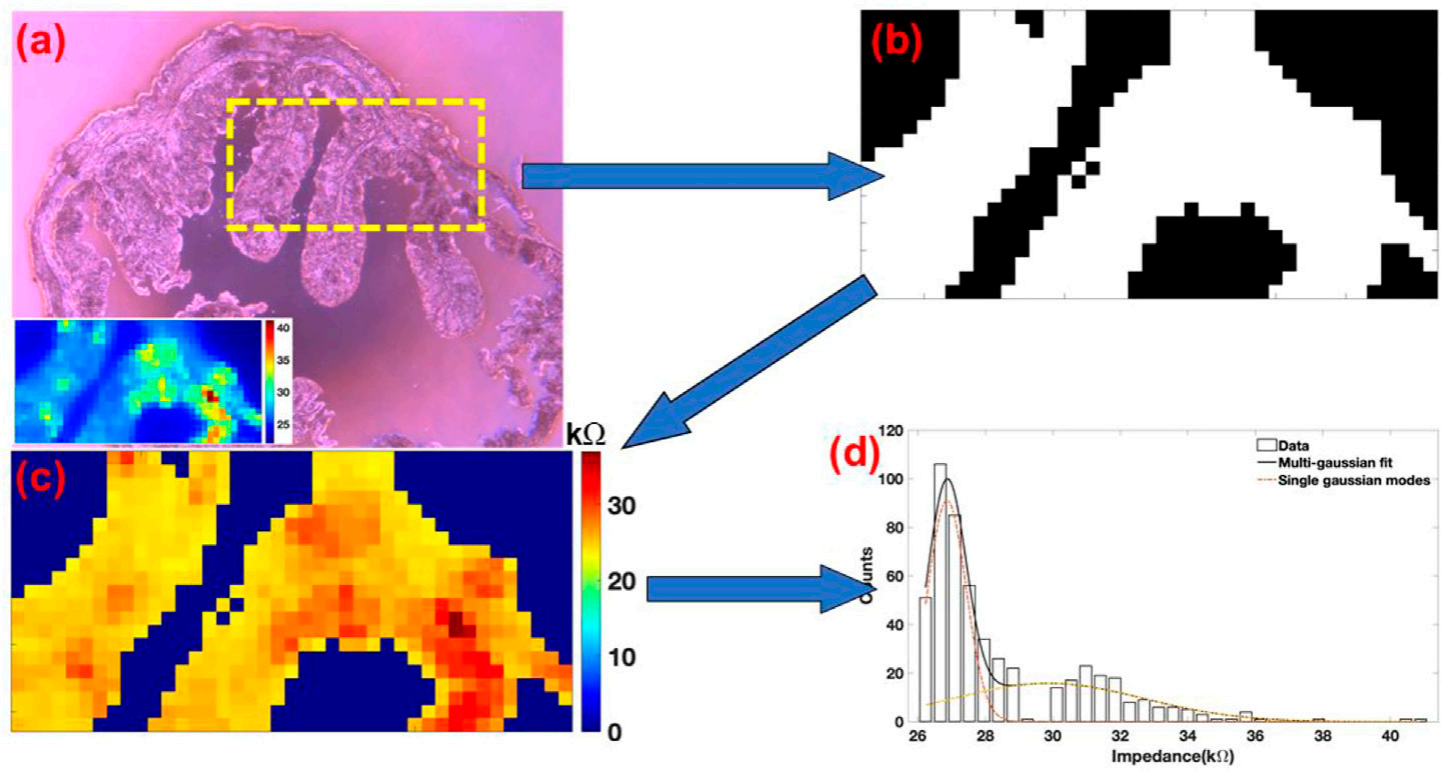
**(A)** Bright field image of the colon section. A dotted yellow rectangle marks the region of the scan. Bottom left a representative impedance map of the image. **(B)** Binary image of the colon section used as an overlay for masking the glass substrate. **(C)** Masked image of the colon section. Data from the surrounding glass was then selectively removed. **(D)** Representative impedance data arising exclusively from the colon tissue section was further plotted as a histogram.

**FIGURE 4:**
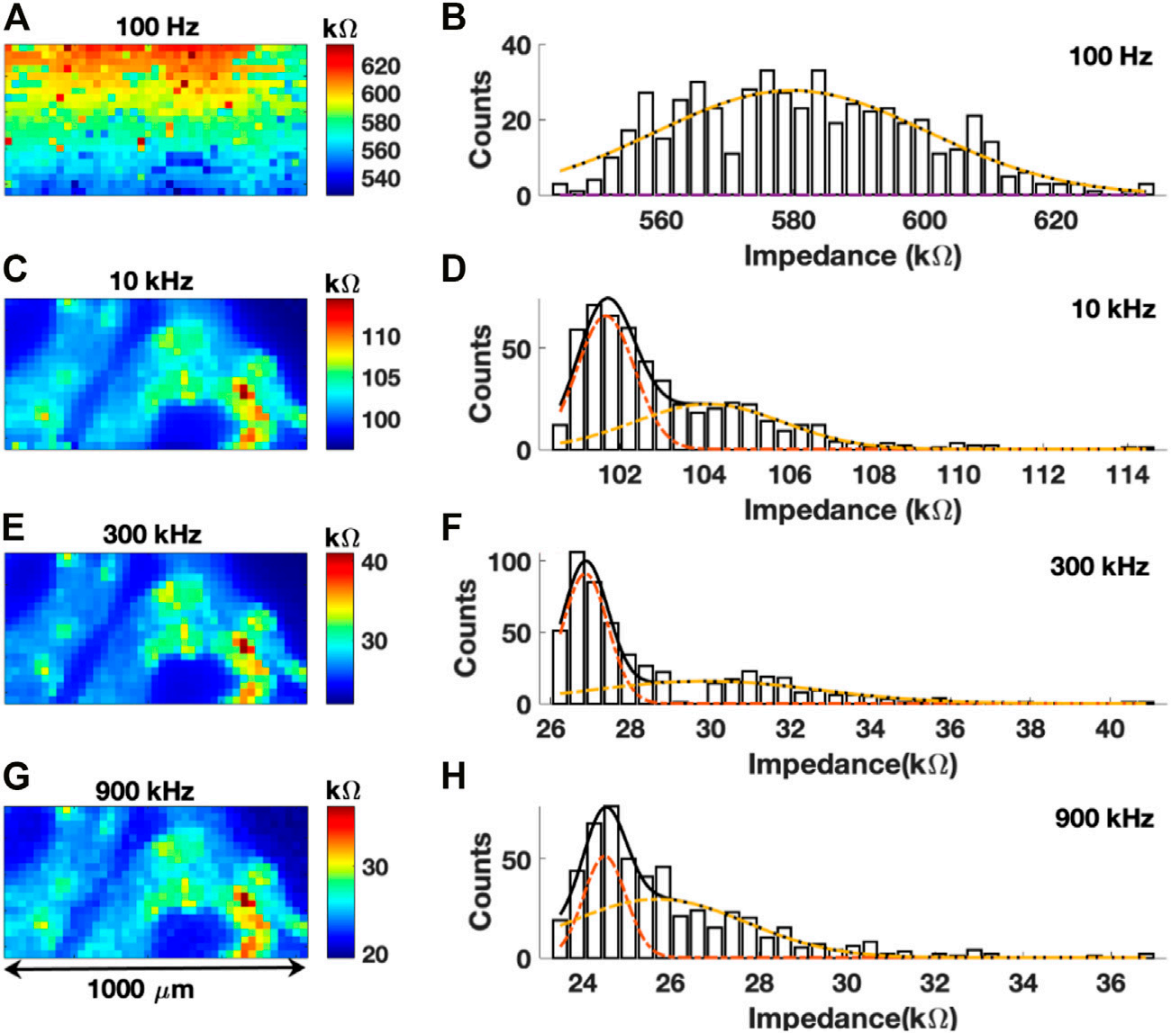
**(A,C,E,G)** Impedance map of colon sections at frequencies of 100 Hz, 10 kHz, 300 kHz, and 900 kHz, respectively. **(B,D,F,H)** Histogram of impedance values with a Gaussian fit for 100 Hz, 10 kHz, 300 kHz, and 900 kHz scans, respectively.

Impedance scanning of the colon section was performed at frequencies of 100 Hz, 10 kHz, 300 kHz, and 900 kHz. Analysis of the impedance maps at different frequencies revealed significant differences in the scan contrast (Figure 4). To isolate signatures from the tissue with respect to the surrounding amorphous SiO_2_ surface (glass slide), a binary mask was used as an overlay. Here, the bright field image of the colon section being subjected to multi-frequency impedance analysis can be observed in Figure 3A. The scan area was 1,000 μm × 500 μm and is indicated by a dotted yellow rectangle. An inset on the bottom left shows a representative impedance map of the region. Visual examination of 300 kHz scan (Figure 4E) shows minimum overlap intensities between the CCS and the glass surface. Using this as a reference image, 25 kΩ was used as a threshold to develop a binary mask shown in Figure 3B. This mask was overlaid over all the four images to remove the impedance values of the surrounding glass surface. Figure 3C shows the mask overlaid image of the 300 kHz scan. Impedance data of the surrounding glass was removed from the image and the rest of the impedance data in the image from the CCS was examined as a histogram. Finally, histograms from the four images were fitted with the Gaussian distribution function as shown in Figure 3D.

Representative impedance maps obtained at four different frequencies over the same region of the colon tissue and their respective histograms are shown in Figure 4. Imaging was performed with a 25 μm Pt microelectrode with a spatial resolution of 30 μm . An initial scan was performed at 100 Hz, then at 10 kHz, and thereafter at 300 kHz and 900 kHz. These frequencies cover the low, mid, and high frequency ranges, incorporating the α and β dispersions regions of the spectra. The duration of each scan was approximately 1 h and 15 min.

The impedance map from the first scan at 100 Hz is shown in Figure 4A. No identifiable histological features of the colon tissue can be observed from the image. Due to high tissue permittivity at 100 Hz, no contrast or different electrochemical signatures were observed within the colon tissue. The polarization resistance was also very high which interfered with ionic diffusion processes with respect to the local surface chemistry of the tissue. A uniform distribution of intensities could also be observed in a unimodal Gaussian fit of the histogram of impedance values, with a maximum impedance of ∼580 kΩ (Figure 4B). Critically, impedance mapping at this frequency revealed no spatial information regarding the biochemical composition of the colon tissue. Conversely, a subsequent scan of the same region with a frequency of 10 kHz revealed significant details related to the histology of the colon tissue (Figure 4C). The lamina propria has a mean impedance of 101.68 kΩ. Conversely, few isolated regions in the sub-mucosa and circular muscles have impedance greater than 104.09 kΩ. The same bimodal distribution of impedance values was also observed (Figure 4D).

At 300 kHz (Figure 4E) the image quality was slightly improved as the boundaries of the lamina propria do not overlap with the surround glass substrate. The same can be observed from the histogram of impedance values in Figure 4F. The mean value of impedance of the lamina propria region was 26.86 kΩ. At 900 kHz (Figure 4G), one can observe relatively poorer contrast with intermixing of intensities between the tissue and surrounding glass substrate. The resulting histogram in Figure 4H shows poor contrast between the two distributions of impedance. Table 1 shows the difference between two Gaussian peaks at 10, 300, and 900 kHz. In Figures 4D, F, H, first distribution plot given in Table 1 is marked by a dotted red line, and the second distribution plot given in Table 1 is marked by a dotted yellow line. Mean (µ) and standard deviation ( $\left.\sigma \right)$ of both the Gaussian peaks are given in the second and third column of Table 1. Separation between the mean values of both distributions of impedance plots is given in the fourth column of Table 1. The segregation between higher and lower intensities was observed to be most pronounced at 300 kHz and it can be inferred that these mid range frequencies are more suitable for differentiating between local biochemistry in complex tissues with a DMEM electrolyte. When SECM imaging is performed in DC-mode, previous approaches Razzaghi et al. (2015) have tried to improve the contrast of non-conductive biological materials by using conductive indium tin oxide (ITO) as a substrate for imaging. With better segregation of intensities at certain frequencies, contrast enhancement in biological samples can be achieved. This can help in describing microscale heterogeneities in complex biological samples with more accuracy.

**TABLE 1:** Mean $\left(\mu \right)$ values with standard deviation $\left(\sigma \right)$ of the first and the second Gaussian peaks and difference in kΩ between the two Gaussian peaks.

Scan frequency (kHz)	First Gaussian (FG) ( μ ± σ)	Second Gaussian (SG) $\left(\mu \;\pm \;\sigma \right)$	Difference (SG – FG)
10	101.68 ± 0.29 kΩ	104.09 ± 0.74 kΩ	2.42 ± 0.80 kΩ
300	26.86 ± 0.24 kΩ	29.87 ± 1.20 kΩ	3.0 ± 1.22 kΩ
900	24.50 ± 0.21 kΩ	25.7 ± 0.85 kΩ	1.19 ± 0.88 kΩ

A gradual decrease in impedance can be observed in frequency versus impedance spectra (Supplementary Figure S2B) with increasing frequency. A similar gradient of impedance values could be seen from 100 Hz to 900 kHz scans. Specifically, the median impedance value of the image scanned at 100 Hz was ∼580 kΩ which decreased to ∼30 kΩ at a scan frequency of 300 kHz. With increasing frequency, both the sample polarization resistance and polarization capacitance increase, and current flow is regulated by reaction kinetics and the diffusion of reactive components towards and away from probing electrode (Schwan, 1968). With high frequency (900 kHz) scans, the shielding effect arising from an electrostatic double layer is reduced, but ultrafast solvation and friction induced by ionic motion in an electrolyte under an oscillating electric field can give rise to errors in the measurement of impedance values (Chandra et al., 1993; Chandra and Bagchi, 2000). This will also interfere with reaction kinetics between these ions in the electrolyte and the microelectrode.

**FIGURE 5:**
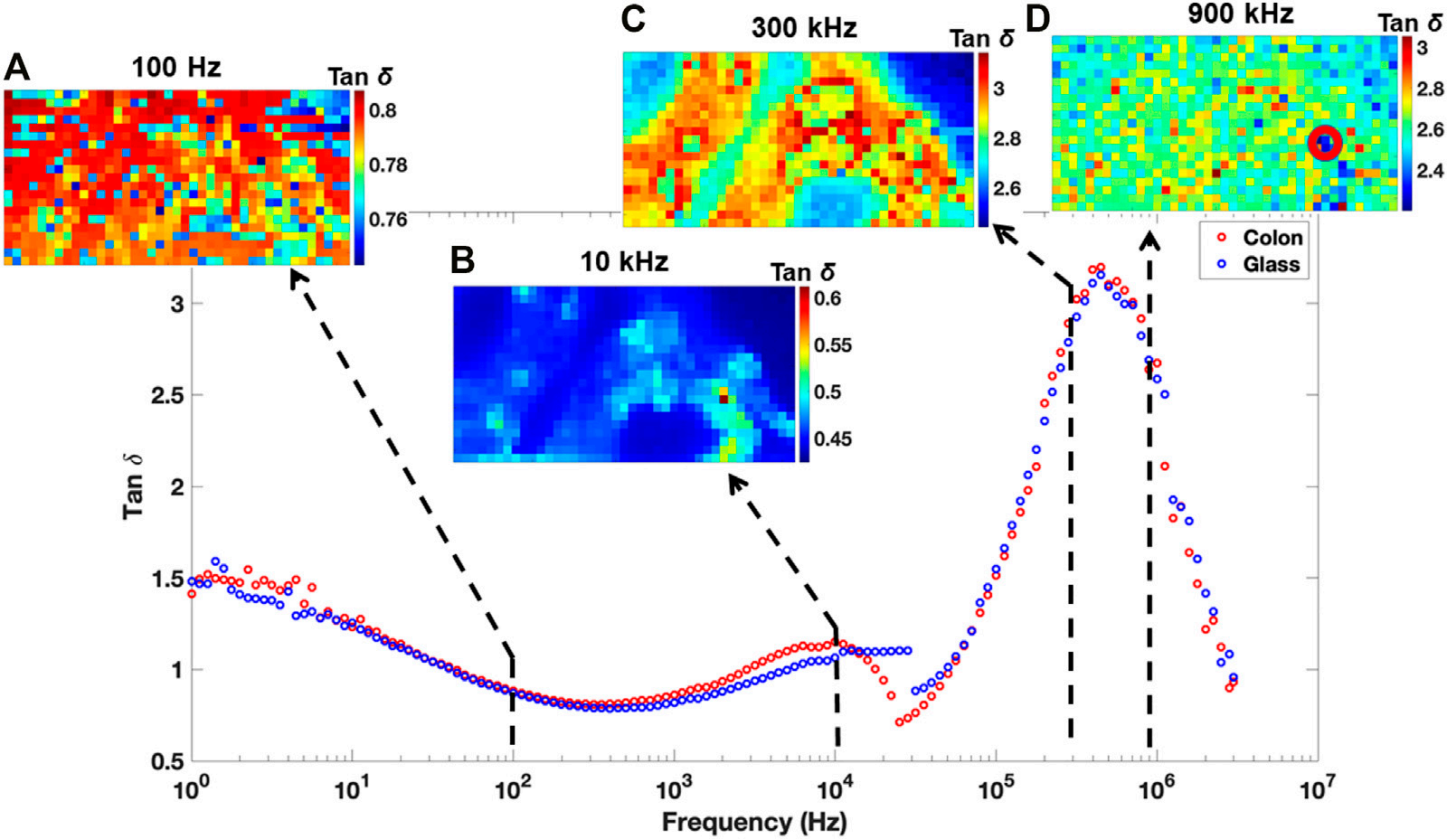
Loss tangent maps at 100 Hz **(A)**, 10 kHz **(B)**, 300 kHz **(C)**, and 900 kHz **(D)**, frequencies superimposed onto a representative frequency vs. tan δ profile of micro-sectioned colon tissue. The position of the probe during the frequency sweep is marked with red circle in the 900 kHz scan.

Subsequent dielectric loss map of colon tissues at frequencies of 100 Hz (Figure 5A), 10 kHz (Figure 5B), 300 kHz (Figure 5C), and 900 kHz (Figure 5D) were developed as described previously, as a ratio of ReZ and ImZ maps. Both real and imaginary maps at different frequencies are given in Supplementary Figure S4. Analysis performed at all frequencies indicates a distinct dielectric signature with respect to the microstructures of the sectioned tissues. At 100 Hz and 900 kHz, one cannot distinguish between glass and tissue surface.

The morphology of the colon section was only identifiable with tan δ maps obtained with scan frequencies of 10 kHz (Figure 5B) and 300 kHz (Figure 5C). An ∼3 fold increase in dielectric loss was also noted in tissues scans at 300 kHz as compared to 10 kHz scan. This increase of tan δ can be attributed to an increasing (becoming less negative) imaginary impedance of tissues when scanned above the 10 kHz frequency range. A gradual increase in the dielectric loss of colon tissues between 100 kHz and 1 MHz frequency range was also observed in the frequency vs. tan δ profile (Figure 5). A closer inspection of the 10 kHz impedance map (Figure 4C) and the tan δ map (Figure 5B) revealed similar regions of dielectric loss with respect to the glass substrate 5(b). A significant increase in dielectric loss can be observed in the colon section relative to the underlying glass substrate at the frequency of 300 kHz giving rise to tan δ values of ∼2.7 (Figure 5C). Here it is important to note that inorganic material like glass [amorphous silicon dioxide (SiO_2_)] will have relatively lower dielectric loss as compared to complex tissues and this will hold true at all the frequencies in the 1 Hz to 1 MHz range. This SiO_2_ surface has also acted as a good reference material when comparing frequency dependent shifts in intensities over the tissue sample. During such complex measurements having a control material can help in identifying any ambiguities during the scan which may affect the consecutive scans at different frequencies over the same region of interest.

During an EIS measurements, a conventioanl 2D frequency vs. tan δ profile gives an averaged electrical signature from the whole tissue. On the contrary, multifrequency maps as shown in Figure 5 in the *β* dispersion range, add another dimension to the information that can be derived from the tissue. Here, heterogenities in tan δ signatures along the whole length of the tissue and different sections of the tissue can be compared. This may not be completely non-invasive but tissue biopsies can be easily examined in a similar manner. With this approach, one can more accurately assess progression of any disorder or extent of damage within a tissue.

Gramse et al. (2013) performed EFM measurements of dipalmitoylphosphatidylcholine (DPCC) lipid bilayer biomembranes with a platinum microprobe to measure the dielectric constant of the lipid bilayers. In their investigations, they were able to demonstrate that such lipid bilayers have an effective dielectric constant of ∼3.2, which was due to the presence of polar head groups in the lipid bilayers. Proteins are also highly polarized molecules and their specific dielectric constants are usually greater than ∼2.5 (Pethig, 1984; Bibi et al., 2016). With 8 μm thin sections, various elements forming the tissue such as lipids, proteins, and other intracellular membrane organelles are exposed to the electric field. The observed high tan δ values of thin colon sections with scanning frequencies >10 kHz may also be due to the uniform distribution of lipids in this tissue and it is well established that high dielectric losses can be observed in materials possessing a high dielectric constant (Zhu, 2014). Another possible explanation could be that the frequency of 300 kHz is closest to the peak values of tan δ of various biomolecular components of this tissue, which results in regions of high dielectric loss across the tissue. Due to this very reason, no significant overlap in tissue signatures as compared to the surrounding glass surface was observed at 300 kHz. During imaging in cell culture media, some biological material may adhere to the surrounding glass surface. This can give similar electrical signatures on tissue and surrounding glass surface at the frequency of 10 kHz. But lipid content and exposed membrane proteins should give a distinct electrical response with respect to the surrounding glass surface. This effect was observed at the frequency of 300 kHz where any interference from non-specific biomolecular material on both the glass surface and in the tissue was significantly reduced. Therefore mapping a tissue only at one frequency may not be the correct approach when the goal is to identify tissue specific signatures. This technique of SLTM with multifrequency mapping of the tissue can be used to measure local changes in the lipid composition of colon tissues, such as those occurring in colitis and colorectal cancer (Pakiet et al., 2019). With further investigation, it may be possible to isolate the dielectric signatures of various fatty acids that are differentially expressed in various pathological conditions.

Classical SECM imaging is dependent on redox species that are added to the electrolyte. Some of the non-redox proteins that are conductive but when denatured may not be detectable by conventional SECM imaging (Zhang et al., 2020). SLTM can measure minute fluctuations in various types of biomolecules that are sensitive to frequency dependent dielectric variations and which may be identified in the β dispersions region. Histological analysis is typically performed over large area tissue sections. In the same manner, large area scans as discussed in this study can provide crucial insight into electronic properties of diseased and healthy tissues.

## Conclusion

Here we report for the first time the use of loss tangent mapping to image histological sections of tissues and multifrequency measurements for the optimization of high contrast SECM imaging in cell culture media. Previous biological SECM studies have focused on the development of current and impedance maps which may not directly correlate with dielectric fluctuations at various frequencies. SLTM can give detailed insight into perturbations in physiological conditions and minute changes in protein or lipid chemistry. In future studies, it would be interesting to observe whether any modification in local chemistry due to disease or injury can affect the dielectric signature of the region of interest.

## Data Availability

The raw data supporting the conclusion of this article will be made available by the authors, without undue reservation.
