# Supplementary material for: Multifrequency dielectric mapping of fixed mice colon tissues in cell culture media via scanning electrochemical microscopy
Source: Front Bioeng Biotechnol. 2023 Feb 9;11:1063063. doi: 10.3389/fbioe.2023.1063063 (PMC9947134; doi:10.3389/fbioe.2023.1063063)
Supplement: Supplementary file 1 [file DataSheet1.docx]

*Supplementary Material*

**Multifrequency Dielectric Mapping of Fixed Mice Colon Tissues in Cell Culture Media Via Scanning Electrochemical Microscopy**

**Varun Vyas^1,2(🖂)^, Niranjan G. Kotla^1^, Yury Rochev^1^ , Anup Poudel^1^, Manus Biggs^1^**

^1^ CÚRAM, SFI Research Centre for Medical Devices, National University of Ireland Galway, Ireland

^2^Université de Lorraine, CNRS, LIEC, Nancy, France


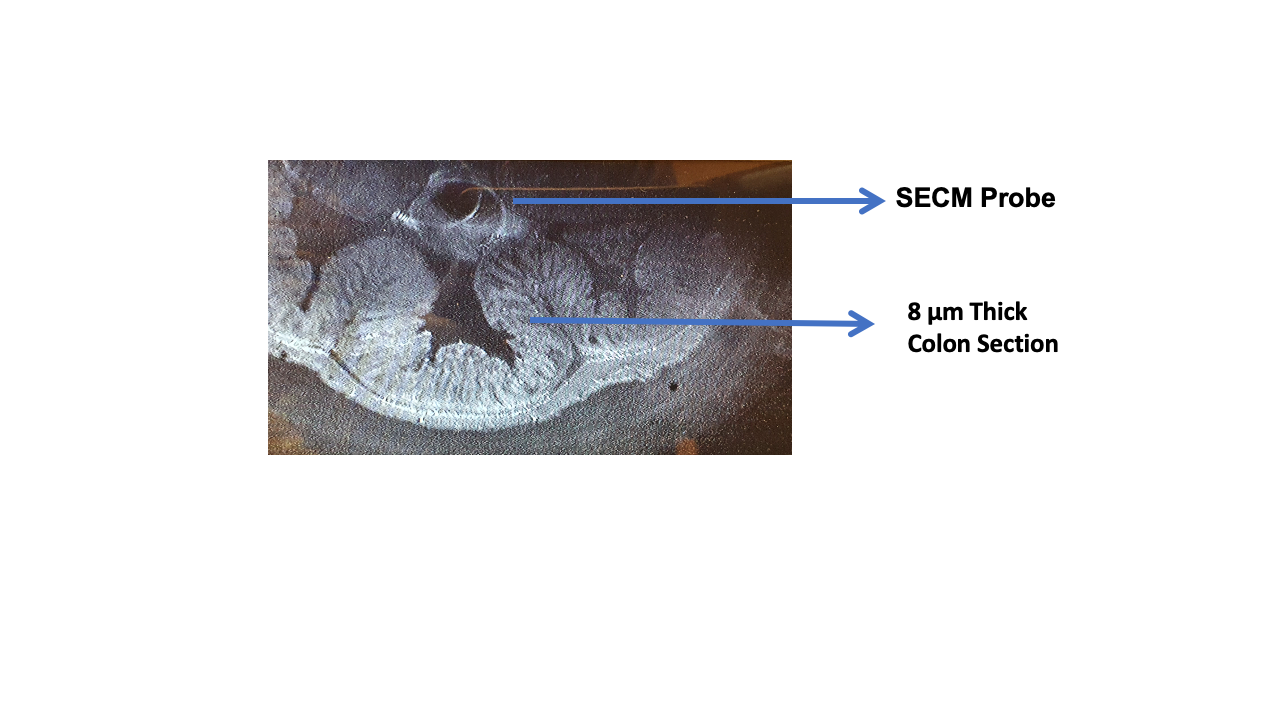


**Supplementary Figure 1. Bright field image of the colon section with 25** $\boldsymbol{\mu m}$ **Pt microelectrode over the lumen region.**


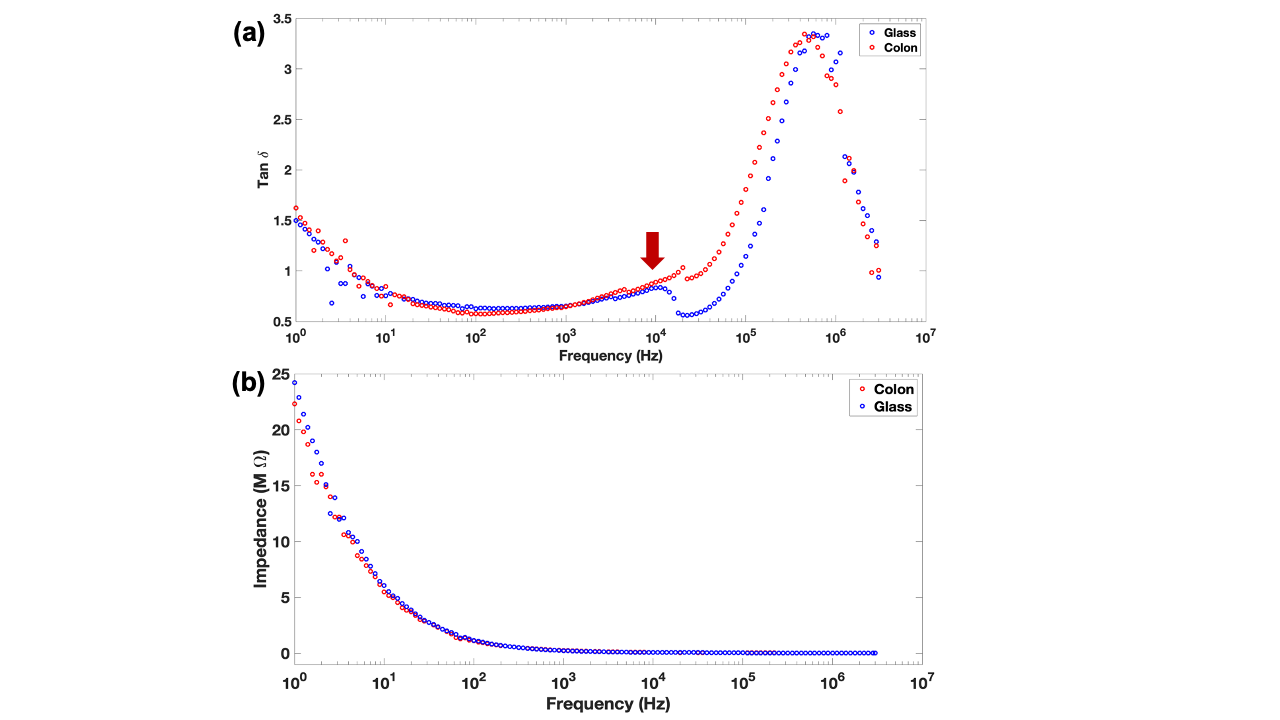


**Supplementary Figure 2. (a) Plot of frequency vs tan** $\boldsymbol{\delta}$**. (b) Plot of frequency vs impedance.**


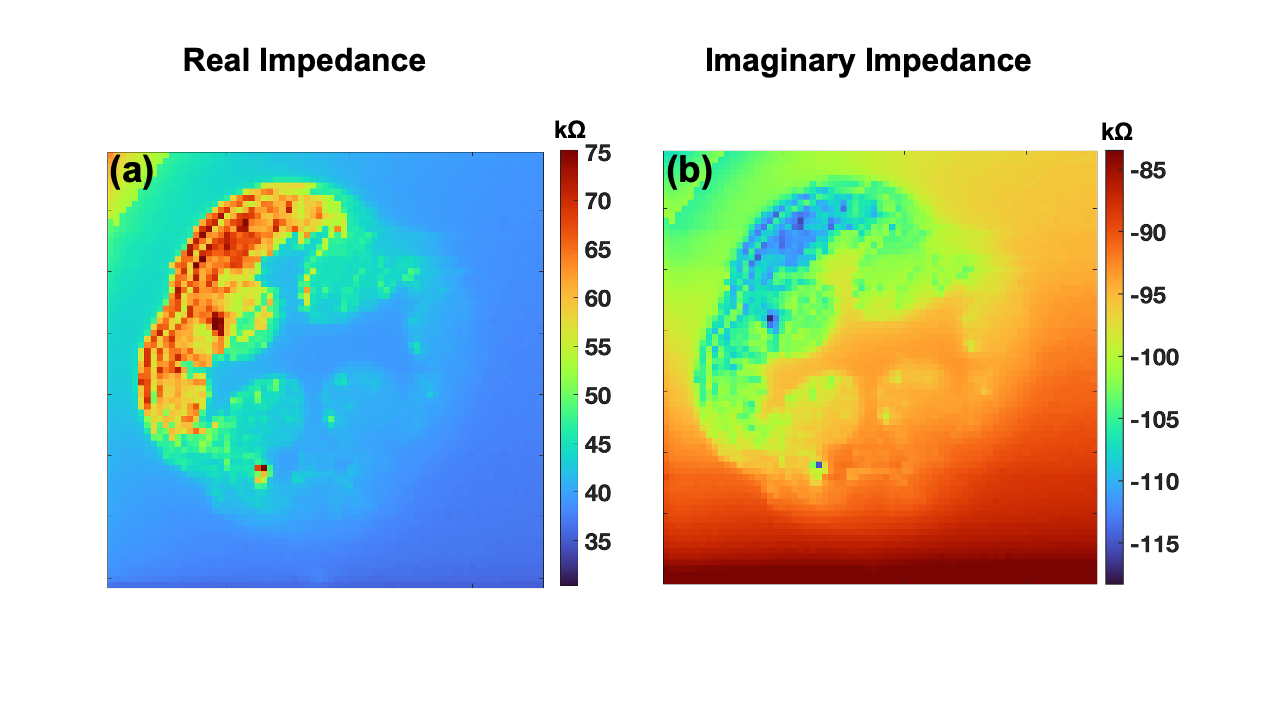


**Supplementary Figure 3. (a) Real impedance map of colon section (b) Imaginary impedance map of colon section.**


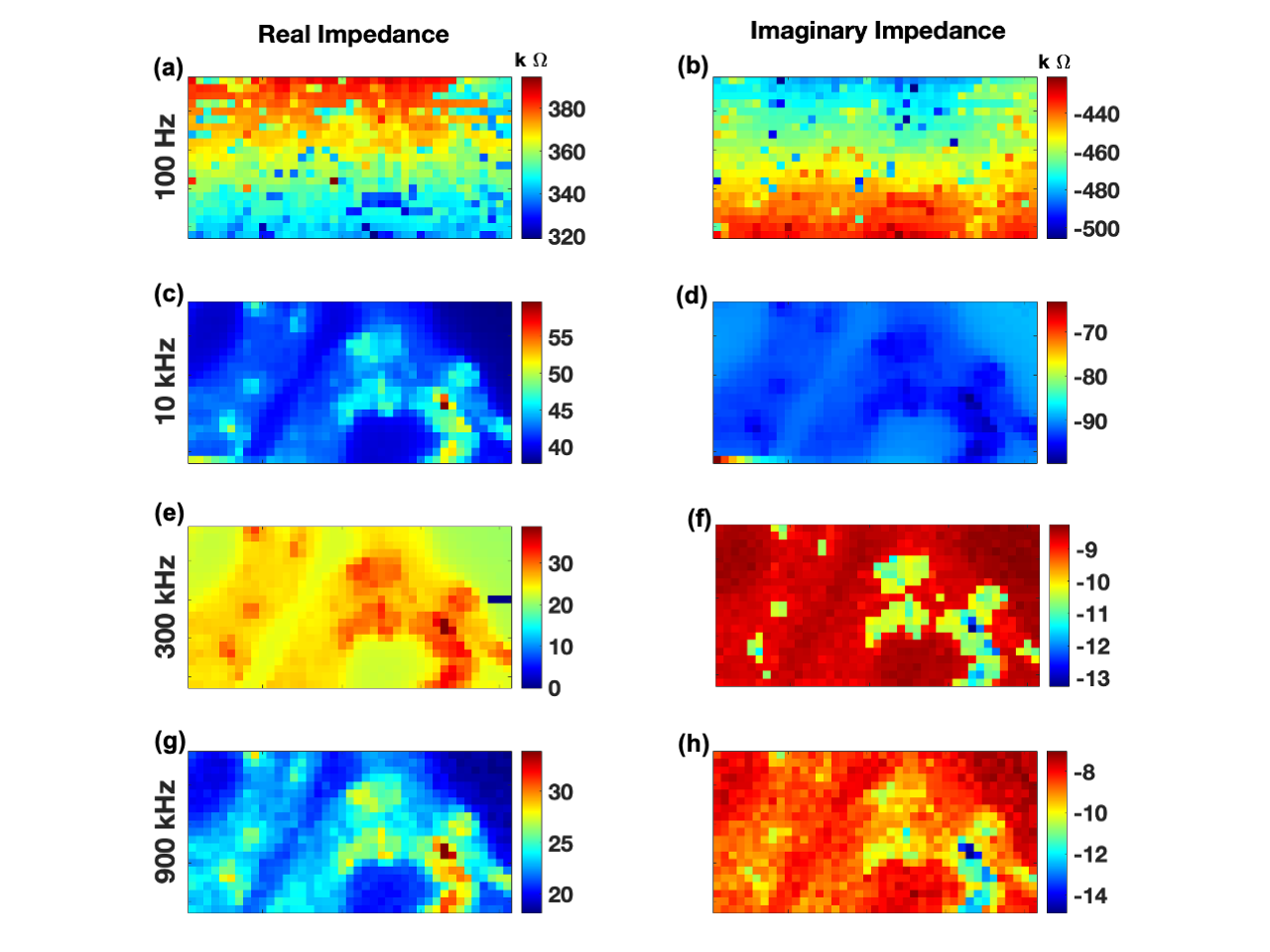


**Supplementary Figure 4. (a) Real impedance map of colon section at 100 Hz (b) Imaginary impedance map of colon section at 100 Hz (c) Real impedance map of colon section at 10 kHz (d) Imaginary impedance map of colon section at 10 kHz (e) Real impedance map of colon section at 300 kHz (f) Imaginary impedance map of colon section at 300 kHz. (g) Real impedance map of colon section at 900 kHz (h) Imaginary impedance map of colon section at 900 kHz.**
